# Leukocyte cell population data as potential markers of COVID-19 disease characterization

**DOI:** 10.5937/jomb0-41589

**Published:** 2023-08-25

**Authors:** Giovanni Introcaso, Arianna Galotta, Laura Salvini, Elena Maria Faioni, Alice Bonomi, Emilio Assanelli, Maria Luisa Biondi

**Affiliations:** 1 Università di Milano, Centro Cardiologico Monzino IRCCS, Unit of Laboratory Medicine, Milan, Italy; 2 Centro Cardiologico Monzino IRCCS, Unit of Biostatistics, Milan, Italy; 3 Centro Cardiologico Monzino IRCCS, Emergency Department, Milan, Italy

**Keywords:** leukocyte parameters, COVID-19, cell population data, emergency department, clinical markers, laukocitni parametri, COVID-19, podaci ćlijske populacije, hitna služba, klinički marker

## Abstract

**Background:**

The usefulness of leukocyte cell population data (CPD) is currently being investigated. In COVID-19 pandemic several reports showed the clinical importance of hematological parameters. Our study aimed to assess CPDs in Sars CoV-2 patients as new disease markers.

**Methods:**

From February to April 2020 (1st wave) 540 and from September to December 2020 (2nd wave) 2821 patients respectively were enrolled. SARS CoV-2 infection diagnosis was carried out by Multiplex rRT-PCR from nasopharyngeal swabs. CPDs were detected by XN 2000 hematology analyzer (Sysmex Corporation). A comparison between two disease waves was performed. Additionally, C-reactive protein (CRP) and lactate dehydrogenase (LDH) were assayed.

**Results:**

CPDs were classified into: cell complextity, DNA/RNA content and abnormal sized cells. We detected parameters increased from the reference population for all cell types for both 1st and 2nd wave (p<0.05). However, in the 2nd vs 1st wave 5 CPDs vs 9 CPDs were found. In addition we observed higher CPD values of the 1st compared to 2nd wave: (NE-SFL) (p<0.001), (LY-Y) (p<0.0001), (LY-Z) (p<0.0001), (MO-X) (p<0.0001), (MO-Y) (p<0.0001). These findings were confirmed by the higher concentrations of CRP and LDH in the 1st vs 2nd wave: 17.3 mg/L (8.5-59.3) vs 6.3 mg/L (2.3-17.6) (p<0.001) and 241.5 IU/L (201-345) vs 195 IU/L (174-228) (p< 0.001) (median, interquartile range) respectively.

**Conclusions:**

CPDs showed increased cell activation in 1st wave patients confirmed by clinical and biochemical data, associated with worse clinical conditions. Results highlighted the CPDs as disease characterization markers or useful for a risk model.

## Abstract (sr)

**Uvod:**

Trenutno se istražuje korisnost podataka o populaciji ćelija leukocita (CPD). U pandemiji COVID-19 nekoliko izveštaja je pokazalo klinički značaj hematoloških parametara. Naša studija je imala za cilj da proceni CPD kod pacijenata sa Sars CoV-2 kao nove markere bolesti.

**Metode:**

Od februara do aprila 2020. (1. talas) upisano je 540 i od septembra do decembra 2020. (2. talas) 2821 pacijenata. Dijagnoza infekcije SARS CoV-2 sprovedena je pomoću Multiplek rRT-PCR iz briseva nazofarinksa. CPD su detektovani hematološkim analizatorom KSN 2000 (Sysmex Corporation). Urađeno je poređenje između dva talasa bolesti. Pored toga, ispitani su C-reaktivni protein (CRP) i laktat dehidrogenaza (LDH).

**Rezultati:**

CPD su klasifikovani na: kompleksnost ćelija, sadržaj DNK/RNA i ćelije abnormalne veličine. Otkrili smo povećanje parametara u odnosu na referentnu populaciju za sve tipove ćelija i za 1. i za 2. talas (p<0,05). Međutim, u 2. protiv 1. talasa pronađeno je 5 CPD-a naspram 9 CPD-a. Pored toga, primetili smo veće vrednosti CPD-a za 1. u poređenju sa 2. talasom: (NE-SFL) (p<0,001), (LI-I) (p<0,0001), (LI-Z) (p<0,0001), (MO-X) (p<0,0001), (MO-Y) (p<0,0001). Ovi nalazi su potvrđeni višim koncentracijama CRP i LDH u 1. protiv 2. talasa: 17,3 mg/L (8,5-59,3) naspram 6,3 mg/L (2,3-17,6) (p<0,001) i 241,5 IU/L (201-345) naspram 195 IU/L (174-228) (p<0,001) (medijana, interkvartilni opseg) respektivno.

**Zaključak:**

CPD su pokazali povećanu aktivaciju ćelija kod pacijenata 1. talasa potvrđenu kliničkim i biohemijskim podacima, udruženu sa lošijim kliničkim stanjima. Rezultati su istakli CPD kao markere karakterizacije bolesti ili korisni za model rizika.

## Introduction

The COVID-19 pandemic remains currently a worldwide health emergency. As it is known, pandemic negatively affected the continuity of care for patients with various diseases. Several studies [1] [2] [3] defined its impact on cardiovascular pathologies as well as on 30-day mortality and in-hospital mortality. There were differences among the first and subsequent waves, as well as between different infections caused by SARS CoV-2 variants. This involved the ability of hospitals in general and specifically for a tertiary cardiologic hospital such as ours to deal with the SARS CoV-2 epidemic, a burden particularly for the emergency department (ED) due to urgent COVID-19-related illnesses as well as cardiovascular diseases [4]. In this setting, along with immunological and molecular tests for virus detection, biochemical and hematological markers could be useful for COVID-19 characterization. Several studies showed leukocyte alterations detected in SARS CoV-2 positive patients, [5] [6] [7] [8], based however on time-consuming blood cytometry investigations, which require specific instruments and dedicated personnel. New hematological analyzers allow to quickly obtain biological information through a routine test panel from peripheral blood analysis. Previous studies suggested that use of leukocyte cell population data (CPD) aids in early diagnosis or in classifying the severity of COVID-19 at hospital presentation [9] [10] [11] [12]. The purpose of our study was to evaluate the leukocyte parameters, using an automated blood analyzer, as potential cellular markers for the disease characterization. For this, a clinical and laboratory comparison of patients admitted to ED during the first and second SARS CoV-2 outbreak was performed.

## Materials and methods

### Study cohort and protocol

**Figure 1:**
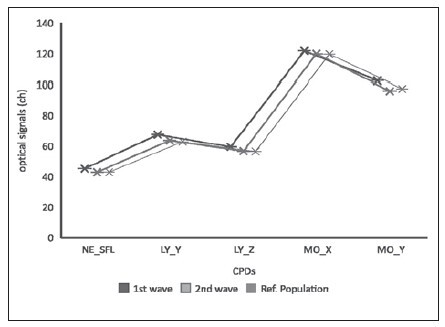
Comparison between leukocyte CPDs from 1st wave to 2nd wave and a reference population. Note: The greatest number of cellular anomalies concerns the DNA/RNA content (see NE-SFL, LY-Y, MO-Y). NE-SFL= fluorescent light intensity of the neutrophil area, LY-Y= fluorescent light intensity of limphocyte, LY-Z= forward scattered light intensity of limphocyte, MO-X= side scattered light intensity of monocyte, MO-Y= fluorescent light intensity of monocyte.

A retrospective and observational study was conducted using the clinical and laboratory database through the automated hematology analyzer Sysmex XN 2000 (Sysmex Corporation, Kobe, Japan) 00-21 software version. From February to April 2020 (1^st^ wave), five hundred forty patients (490 negative and 50 SARS CoV-2 positive), as well as two thousand eight hundred twenty-one patients from September to December 2020 (2^nd^ wave) (2762 negative and 59 SARS CoV-2 positive) were enrolled. The main diagnosis at presentation was a respiratory distress as well as the suspicion of cardiological diseases. Leukocyte parameters were evaluated through a comparison between the COVID-19 positive population (N=50) (1^st^ wave) and (N=59) (2^nd^ wave) versus a reference population (N=490) and (N=2762) respectively. To obtain a more specific control group (reference population N=54), a selection by leucocytes and hemoglobin values through a propensity score method was performed (Figure 1). Afterwards, CPDs of the 1^st^ wave (N=50) and 2^nd^ wave (N=59) were compared and their relationship with biochemical markers was analyzed. The study was approved by the Ethical Committee of the Monzino Cardiology Center and conformed to the principles outlined in the Declaration of Helsinki.

### Hematological tests and the meaning of CPDs

Blood samples were drawn into anticoagulant K3 EDTA tube (Vacutainer BD, UK) and processed within 1 hour along with biochemical testing of routine triage panel. The XN 2000 analytical platform enabled blood cell counts using fluorescent dyes and a specific analytical channel WDF for leukocyte analysis. A software based on hematology rules evaluated scattergrams and analytical flags allowing an accurate automated classification of leukocyte populations: neutrophils, eosinophils, basophils, lymphocytes, monocytes. Leukocyte CPDs, as optical signals are divided into: side scattered light intensity, side scattered light distribution width, fluorescent light intensity, fluorescent light distribution width, forward scattered light intensity, forward scattered light distri bution width. They identify all cellular characteristics of neutrophils (NE), lymphocytes (LY), monocytes (MO) as: cell complexity (LY-X, MO-X, NE-WX, LY-WY, MO-WX), cellular content in DNA and RNA (NE-SFL, LY-Y, MO-Y, NE-WY, LY-WY, MO-WY), abnormal sized cells (NE-FSC, LY-Z, MO-Z, NE-WZ, LY-WZ, MO-WZ). They were reported in arbitrary units of light scattering of channel (ch). A daily quality control, a careful preanalytical check, and external quality evaluation assured a reliable total testing process (TTP).

### Molecular rRT-PCR assay

Molecular assays were carried out using a Multiplex rRT-PCR from samples collected by nasopharyngeal swabs. At the beginning of the pandemic spread, we evaluated three viral genes: E, RdRP, N, to detect SARS CoV-2, according to the manufacturing procedures. Afterwards, two genes: RdRp and ORF8 (SARS-CoV-2 Elite MGB kit) (OSANG Healthcare, Anyangcheondong-ro, Dongan-gu, Anyang-si, Gyeonggi-do, Korea) on ELITech InGenius platform (Torino, Italy) were analyzed.

### Biochemical measurements

Markers C-reactive protein (CRP) and lactate dehydrogenase (LDH) were measured, at the hospital admission of patients, using Atellica Clinical Che mistry Analyzer (Siemens Healthcare GmbH, Erlangen Germany). Measurements were performed on sera samples obtained by centrifugation at 1500 g for 10 minutes.

### Statistical analysis

Statistical analysis was performed using SAS version 9.4 (SAS Institute, Cary, NC). Continuous variables are presented as mean ± standard deviation (SD) and were compared using the t-test for independent samples. Variables not normally distributed are presented as median and interquartile ranges and were compared with the Wilcoxon rank sum test; these variables were log-transformed before any analysis. Categorical variables are expressed as frequency and percentage and were compared using Pearson’s 2 test or Fisher’s exact test, as appropriate. Spearman’s correlation coefficient was calculated to assess the correlation between the variables of interest. Statistical analysis using the univariate and multivariate general linear regressions was carried out; specifically, to evaluate the association between LDH and/or CRP, with the following CPDs: NE-SFL, LY-Y, LY-Z, MO-X, MO-Y, NE-WX, NE-WY, NE-WZ, MO-WX. A propensity score matching for the CPD comparison was used; propensity score was computed using a multivariable logistic regression model using the following variables WBC and hemoglobin. A p<0.05 was considered to be statistically significant.

## Results

### Patients characteristics

During the pandemic outbreaks, we observed different clinical characteristics of the patients admitted to ED, in particular a greater number of SARS CoV-2 pneumonia and severe clinical conditions of patients during the 1^st^ wave compared to the second (Table 1). Indeed, we recorded many secondary diseases with superinfection and three major complications that ended in death. In the 2^nd^ wave there were many SARS CoV-2 infections without pneumonia and no cases of serious complications.

**Table 1:** Main clinical characteristics of COVID-19 patients presented to ED. ACS= acute coronary syndrome, AMI= acute myocardial infarction, AKI= acute kidney injury, AF= atrial fibrillation

	1^st^ Wave<br>(N=50)	2^nd^ Wave<br>(N=59)	P-value
Age (years)	70.6±12	64.2±15.1	0.001
Male sex (%)	66	66	1.000
Primary Illness			
ACS	1 (2.0%)	0 (0%)	0.458
AMI	4 (8.0%)	0 (0%)	0.041
Sars Cov-2<br>pneumonia	38 (76.0%)	25 (42.4%)	<0.001
Chest pain	0 (0%)	1 (1.7%)	1.000
Angina pectoris	0 (0%)	1 (1.7%)	1.000
Sars Cov-2 infection<br>without pneumonia	7 (14.0%)	27 (45.8%)	<0.001
Pericarditis	0 (0%)	1 (1.7%)	1.000
AF	0 (0%)	2 (3.4%)	0.498
AKI	0 (0%)	1 (1.7%)	1.000
Syncope	0 (0%)	1 (1.7%)	1.000

### Leukocyte CPDs analysis

Leukocyte CPDs in both the first and the second outbreak were compared; in a first analysis, we observed statistically significant differences between the CPDs of SARS CoV-2 patients and a reference population of patients admitted to our cardiology ED. Considering the same CPDs, we detected a greater number of CPD alterations in the patients from the 1^st^ wave (Table 2). However, cytometric changes as CPDs concerning lymphocytes, monocytes and neutrophils were also observed in patients from the 2^nd^ wave (Table 2). A further analysis, using a selected reference population (N=54) as described in Methods, confirmed previously differences between cases and controls for the following CPDs: LY-WY(ch) (p=0.003), LY-X(ch) (p=0.017), MO-WZ(ch) (p<0.0001), NE-WZ(ch) (p=0.038).

**Table 2:** Leukocyte CPDs of COVID-19 patients compared to the Reference Population. Note: NE-SFL= fluorescent light intensity of the neutrophil area, LY-X= side scattered light intensity of lymphocyte, LY-Z= forward scattered light intensity of lymphocyte, LY-WX= side scattered light distribution width of lymphocyte, LY-WY= fluorescent light distribution width of lymphocyte MO-X= side scattered light intensity of monocyte, MO-Y= fluorescent light intensity of monocyte, NE-WZ= forward scattered light distribution width of neutrophil, MO-WX= side scattered light distribution width of monocyte, MO-WY= fluorescent light distribution width of monocyte.

	Covid-19 positive<br>(N=50, 1^st^ wave)	Ref. Population<br>(n=450)		Covid-19 positive<br>(N=59, 2^nd^ wave)	Ref. Population<br>(n=2762)	
	Mean±SD	Mean±SD<br>P-value	Mean±SD<br>P-value	Mean±SD	Mean±SD	P-value
NE-SFL (ch)	45.1±3.3	44.1±2.3	0.007	42.8±2.4	43.5±3.6	0.139
LY-X (ch)	82.2±2.1	82.2±2.1	0.026	79.3±2.1	78.8±2.3	0.080
LY-Z (ch)	59.4±1.6	58.6±1.3	<0.001	56.6±0.9	56.8±2.3	0.380
LY-WX (ch)	473.9±58.7	469.9±44.1	0.943	539.2±105.8	515.4±65.8	0.015
LY-WY (ch)	853.9±87.3	875.5±80.8	0.114	832.1±83.7	915.6±207.2	<0.0001
MO-X (ch)	121.7±2.9	120.0±2.0	<0.001	119.7±2.4	120.3±2.9	0.131
MO-Y (ch)	102.4±8.8	99.4±5.5	<0.001	95.8±5.4	99.7±8.8	<0.001
NE-WX (ch)	318.8±25.1	306.8±15	<0.001	318.3±15.5	316.8±22.2	0.610
NE-WY (ch)	635.5±63.0	616.±30.9	0.032	633.9±33.2	647.9±81.1	0.226
NE-WZ (ch)	675.2±48.0	654.7±33	<0.001	682.6±32.9	669.1±41.7	0.013
MO-WX (ch)	256.8±27.0	266.3±25	<0.001	261.3±27.8	263.6±27.2	0.519
MO-WY (ch)	741.8±87.8	728.1±89.3	0.136	700.2±64.6	648.5±62.7	<0.0001

Finally, we compared leukocyte activation markers in patients from the two outbreaks as illustrated in Table 3. The marked elevation of NE-SFL, LY-Y and MO-Y optical values in patients from the first outbreak suggests a cellular activation detected by the increase of DNA/RNA content (Table 3, Figure 1).

**Table 3:** Main leukocyte parameters and CPDs in Covid-19 patients: 1^st^ versus 2^nd^ wave Note: WBC= leukocyte, NEUT= neutrophil, Lymph= lymphocyte, MONO= monocyte, EOS= eosinophil, BASO= basophil. NE-SFL= fluorescent light intensity of the neutrophil area, LY-Y= fluorescent light intensity of lymphocyte, LY-Z= forward scattered light intensity of lymphocyte, MO-X= side scattered light intensity of monocyte, MO-Y= fluorescent light intensity of monocyte, NE-WZ= forward scattered light distribution width of neutrophil, MO-WX= side scattered light distribution width of monocyte.

	1st wave (n=50)	2nd wave (n=59)	
	Mean±SD	Mean±SD	P-value
WBC (x10^9^/L)	8.6±4.1	6.2±2.2	<0.001
NEUT ( x10^9^/L)	6.3±3.5	4.0±2.3	<0.0001
LYMPH ( x10^9^/L)	1.5±1.1	1.5±0.8	0.076
MONO ( x10^9^/L)	0.7±0.3	0.6±0.3	0.085
EOS ( x10^9^/L)	0.06±0.07	0.04±0.0	<0001
BASO ( x10^9^/L)	0.03±0.02	0.02±0.0	<0.0001
NE-SFL (ch)	45.1±3.3	42.8±2.5	<0.0001
LY-Y (ch)	67.4±4.3	63.6±6.4	<0.0001
LY-Z (ch)	59.4±1.6	56.6±0.9	<0.0001
MO-X (ch)	121.7±2.9	119.8±2.4	<0.001
MO-Y (ch)	102.4±8.8	95.8±5.5	<0.0001
NE-WZ (ch)	675.2±48.0	683±0.33	0.327
MO-WX (ch)	256.8±27.0	260.8±27.8	0.458

### Biochemical markers

We evaluated in the same COVID-19 patients (1^st^ and 2^nd^ wave), CRP and LDH which showed the following results: CRP (mg/L) 17.3 (8.5–59.3) and 6.3 (2.3–17.6) (P<0.0001); LDH (IU/L) 241.5 (201–345) and 195 (174–228) (P<0.001), median (interquartile range) respectively. An analysis of the correlation among CPDs and biochemical values showed a significant association: in the 1^st^ wave LY-Y with LDH (P=0.046), in the 2^nd^ wave NE-WY with CRP (P=0.015). In addition, a significant association between LDH and CRP values in patients from both the 1^st^ and 2^nd^ wave was identified P=0.001 and P=0.046 respectively. The greater concentration of the CRP and LDH markers in patients from the 1^st^ wave confirmed a worse metabolic and inflammatory state.

## Discussion

Previous studies suggested the clinical usefulness of leukocyte CPDs and their possible application in infectious diseases [9] [13] [14] [15]. Although the involvement of leukocytes in the immune response, particularly monocytes and lymphocytes as immune and adaptive cells, is well known, their clinical application remains not fully established. Leukocyte changes are also extensively described in COVID-19 disease [16] [17] and their interaction with platelets plays a pivotal role in the hemostatic system and in the thromboinflammation complications [17]. In a previous work, we highlighted the key role of platelets that with leukocytes are the first responders of innate immunity in the early phases of COVID-19 infection [18]. Hereby, we conducted a study on leukocyte parameters to suggest their potential use as clinical markers for COVID-19 disease characterization. In a preliminary evaluation, we found significant elevation of CPDs compared to a reference population of patients admitted to our cardiology ED. Results were confirmed by clinical data and biochemical markers LDH and CRP both in the 1^st^ wave and 2^nd^ wave. Of note, CPDs as optical signals such as light distribution width (for example LY-WY, LY-WX, NE-WY) had a high dispersion index and overlapping values in the two waves (Table 2). To the contrary, CPDs of scattered light intensity (for example MO-Y, MO-X, LY-Z) seem to have smaller dispersion. In fact, the comparison of CPDs in the patients belonging to the 1^st^ versus 2^nd^ wave, showed significant differences (Table 3). These CPDs based on fluorescent light intensity NE-SFL, LY-Y, MO-Y detected the higher differences between the two waves, suggesting a greater leukocyte activation in the patients of the 1^st^ wave. Consequently, according to our biochemical and clinical data, NE-SFL, LY-Y and MO-Y represent the leukocyte CPDs detected by a Sysmex platform most altered during Sars Cov-2 infection. These analytical alterations mainly suggest an increase in the cellular DNA/RNA content intended as a metabolic response to organ impairments and disease severity. In this way, based on the experimental and clinical results, they may be useful for the characterization of COVID-19. In agreement with these findings, studies on bacterial infections demonstrated that NE-SFL and MO-X showed the most relevant CPDs in predicting sepsis [14] [15]. In the present study, we highlighted the possible diagnostic significance of some CPDs also in the COVID-19 as showed by cellular sequelae of viral infection. However, we admit also some limitations of our study. CPDs are expected to have a specific cut-off or decision threshold and have not been provided by the manufacturer as parameters for research purposes. Since ours is a tertiary referral hospital for cardiac disease, our control group, were patients admitted to ED with the suspicion of cardiovascular disease then a specific population. Therefore, it remains to design further studies based on a general reference population. In addition, a technological effort allowing an analytical standardization of leukocytes parameters is warranted. In conclusion, leukocyte CPDs revealed significant cellular changes showing leukocyte activation during a viral infection and particularly during SARS CoV-2 infection, still a major worldwide problem, probably due to become endemic. Importantly, as well as other biomarkers, leukocyte parameters could be adopted in predictive models to help guide decision making [19] [20] [21]. Of note, these cell activation markers may add insight into disease severity and help initiate timely treatment. Furthermore, the new discoveries in understanding cellular alterations during viral infection may be remarkable for addressing further future pandemic scenarios.

## Dodatak

### Acknowledgements

We thank our colleagues and the technical staff for their professional support and effective collaboration.

### Contributors

GI conceived the study, conducted the evaluation of data, and wrote the first draft of the manuscript. AG analyzed data. AB analyzed data and involved in the conceptualization of the work. LS and EA collected clinical data. EMF and MLB involved in the revision of the manuscript. The authors edited and approved the final version of the manuscript.

### Funding

This research was supported by the Italian Ministry of Health-Ricerca Corrente to Centro Cardiologico Monzino IRCCS. Italian Research Project n, Grant/Award Number: R1312/-CCM 1380.

### Conflict of interest statement

All the authors declare that they have no conflict of interest in this work.
